# Psychological need fulfillment in virtual teaching: insights of residents and faculty

**DOI:** 10.5116/ijme.6488.2625

**Published:** 2023-06-22

**Authors:** Oksana Babenko, Shannon Gentilini, Nathan Turner, Olga Szafran, Sudha Koppula

**Affiliations:** 1 Department of Family Medicine, University of Alberta, Canada

**Keywords:** Virtual teaching, psychological needs, self-determination theory, medical education, resident, faculty

## Abstract

**Objective:**

To explore benefits and challenges experienced by residents and faculty when teaching in virtual settings.

**Methods:**

This was a qualitative descriptive study employing one-on-one semi-structured interviews with 10 residents and 12 faculty in the Department of Family Medicine at the University of Alberta, Canada, from May 2021 to May 2022. Participants were recruited via social media, resident and department events and email lists. Interview transcripts were analyzed descriptively and thematically employing the Self-Determination Theory (SDT) framework to map the identified benefits and challenges as facilitators and barriers to fulfilling teacher’s basic psychological needs for autonomy, competence, and relatedness in virtual settings.

**Results:**

Resident and faculty participants used virtual technology not only to deliver education, but also leveraged various platform features to support their needs in virtual settings. The emerging themes within benefits and challenges of virtual teaching were amenable to mapping onto three basic psychological needs of the SDT framework – autonomy (e.g., increased accessibility; lack of control over teaching environment), competence (e.g., increased self-confidence; technological limitations hindering skill development), and relatedness (e.g., timely exchange of information; difficulty with professional identity formation).

**Conclusions:**

Despite the inherent challenges, teaching in virtual settings can support teachers’ psychological needs. Recommendations for the future delivery and facilitation of virtual learning include: giving high priority to engagement and active participation; nurturing autonomy and greater individual responsibility for learning; and creating an environment of emotional support. The SDT-informed strategies shown to be effective in in-person teaching need to be examined for their applicability in virtual settings.

## Introduction

Teaching the next generation of medical professionals is an integral role of physicians. Through formal curricula and experiential opportunities, postgraduate medical programs across North America prepare resident physicians for their ensuing role as teachers. Until recently, such activities took place predominantly in person. With the rapid onset of the COVID-19 pandemic and the resultant restrictions, teaching activities pivoted to virtual settings and became the only viable option for residents and clinician educators during the pandemic.

The move to virtual teaching during the COVID-19 pandemic relied heavily upon the availability of skilled teachers and their willingness to embrace existing best practices in distance education.^1^ However, much of what was known about best practices and learning outcomes was informed by the research conducted in in-person teaching settings. Some of the best practices, including careful review of weekly feedback to fine-tune sessions, practice scenarios to consolidate learning, and session standardization with quality assurance by senior faculty, were also recommended in virtual settings during the COVID-19 pandemic.^2^

Previous studies have largely examined virtual teaching from the perspective of inferiority/superiority relative to traditional in-person teaching, comparing a variety of teaching strategies and learning outcomes (e.g., performance).^3^^-^^8^ The predominant theme of published studies is that virtual teaching is regarded as more challenging than traditional in-person teaching, with inconclusive findings regarding the degree of improvement in learning outcomes achieved in virtual settings.^3^^-^^6^ More important, the focus of the research was largely on cognitive aspects such as teaching strategies for better student engagement. Non-cognitive aspects (i.e., feelings, motivations, needs) of teaching in virtual settings remain less understood. Virtual teaching in medical education is expected to flourish in the future,^3^ hence, a deeper understanding of teaching in virtual environments is needed going forward. We aim to present here the insights gained from novice (residents) and experienced teachers (faculty) regarding teaching in virtual settings.

This was part of a larger study, the purpose of which was to identify and describe opportunities available for residents’ teaching skill development from the perspective of residents and faculty. Because the data collection coincided with the COVID-19 pandemic, the move to virtual settings presented a unique opportunity. The primary objective of this study was to explore benefits and challenges experienced by residents and faculty when teaching in virtual settings. A subsequent objective was to classify the identified benefits and challenges as facilitators and barriers to teaching in a virtual environment, which can, respectively, support or undermine teacher’s psychological needs in virtual settings.

## Methods

### Study design

This was a qualitative descriptive study employing one-on-one semi-structured interviews with residents and faculty in the Department of Family Medicine at the University of Alberta, Canada. Qualitative interview methodology was used to obtain the perspectives of residents and faculty about virtual teaching experiences and to encourage two-way communication between the participant and the interviewer. Residents were recruited from the urban and rural streams of the two-year family medicine residency program. Faculty were family physician teachers with academic appointments in the department. Residents and faculty were sent a recruitment notice by email, which included a brief study description and invitation to participate. Recruitment notices were also posted on the residents’ association Facebook page and distributed at organized resident and department events (e.g., Resident Academic Day, Family Medicine Grand Rounds, Department Business Group Meeting). Those interested in taking part in the study were requested to contact the study coordinator (SG) for more detailed information and were provided a study information letter. We recruited 10 residents and 12 faculty members. The number of study participants was not predetermined but depended on when data saturation, or the point at which no new information emerged from interviews, was reached. Table 1 shows participants’ demographic information in terms of gender, age, program stream, and for residents, year in the residency program. The study was approved by the University of Alberta Research Ethics Board (Pro00099314). All participants provided verbal informed consent prior to participating in the interview.

**Table 1:** Demographic information for the study participants

Variable		Residents n (%)	Faculty n (%)
		n=10	n=12
Gender			
	Female	7 (70)	6 (50)
	Male	3 (30)	6 (50)
Age			
	20-29 years	8 (80)	-
	30-39 years	2 (20)	4 (33)
	40-49 years	-	2 (17)
	50-59 years	-	3 (25)
	60+ years	-	3 (25)
Residency stream			
	Urban	8 (80)	10 (83)
	Rural	2 (20)	2 (17)
Year in residency program			
	Year 1	6 (60)	-
	Year 2	4 (40)	-

### Data collection

Separate interview guides were developed for the resident interviews and faculty interviews. The interview guides included questions and prompts to encourage discussion. For the larger study, the interview questions inquired about the teaching activities experienced by residents throughout their two-year residency training. The questions examined the setting in which the teaching occurred, who the learners were, and the topics taught. Given the transition to virtual teaching during the COVID-19 pandemic, all participants were also asked about their experiences teaching in virtual settings and associated benefits and challenges (Appendix). All interviews took place between May 2021 and May 2022. The interviews were conducted virtually by the study coordinator (SG) using the Zoom platform to comply with protocols and restrictions mandated during the COVID-19 pandemic. Participants were informed of the study purpose, guaranteed confidentiality, and could withdraw from the study at any time during the data collection phase. Resident participants received a $20 gift card as a token of appreciation for their participation in the study. Audio recorded interviews were between 45 and 60 minutes in duration and were transcribed verbatim by a professional transcription service. Transcriptions were subsequently cleaned by the study coordinator to remove any identifying information and assigned an alphanumeric code for confidentiality and anonymity.

### Data analysis

Descriptive and thematic qualitative analysis methodologies were employed using an iterative coding approach. The research team drew from a wide range of expertise in family medicine, residency training, medical education, and qualitative research. Each member of the research team reviewed the transcripts independently, then met together to discuss and develop a coding template in an iterative manner until no new themes emerged. The initial list of codes was modified through successive readings of transcripts until a full description of the data was obtained. After coding was completed, transcripts were further reviewed collectively to identify emerging themes.

In seeking to understand the themes, specifically the benefits and challenges of teaching in virtual settings, we discovered that the themes that arose from the data aligned with the existing framework of Self-Determination Theory (SDT).^9^^,^^10^ SDT proposes that people have three innate or basic psychological needs: autonomy, competence, and relatedness. Autonomy is the need to have control over choices and actions. Competence is the need to experience mastery. Relatedness is the need for belonging and caring relationships. If satisfied, these psychological needs promote or facilitate an individual’s optimal functioning, growth, and well-being. In contrast, unmet psychological needs can undermine or hinder an individual’s functioning and well-being.^9^^-^^11^

The emergent nature of qualitative research supports the use of a theoretical framework in the analysis stage.^12^ Application of the SDT framework helped us to make sense and structure the organization of the data, facilitate the interpretation of the findings, and increase their applicability. If we had identified a framework prior to data collection, then we may have forced certain preconceptions at the time of interviews and data analysis. Throughout the process of data analysis, we addressed the influence that we as individual researchers had in shaping the data findings through regular peer-debriefing sessions with all the members of the study team. These sessions facilitated reflexivity and reflection among the team members on how our own personal views and predispositions could influence the study and the findings. This also allowed for different perspectives to be heard and any biases to be addressed.

## Results

Benefits and challenges of virtual teaching were identified and subsequently mapped onto the three psychological needs of the SDT framework – autonomy, competence, and relatedness (Table 2).

### Impact on autonomy

Participants appreciated the variety of virtual technology that allowed them to continue with the residency curriculum and teach during the pandemic. They acknowledged the safety benefits to reduce the spread of illness and reported that the use of virtual platforms helped save time and money and provided ease of access to teaching, especially for those residents and faculty working in remote and/or rural areas.

“…you don’t have to take into account commute time in the morning… so I feel like doing it virtually helps people get a bit more rest and possibly like, eat an extra good breakfast that day of the week...” [Resident 6]

“…online has given it some flexibility. They could be anywhere, and they can teach. But when it was in person, you know, there was always that struggle with scheduling residents for enough sessions and then around their schedule because they might be doing their rural rotation and they’re not in town to be able to lead a session.” [Faculty 1]

“For anybody who needed to be in isolation for the pandemic, it used to be that you would just miss the session because they’re in person, but now you don’t. You can just sign in virtually and participate as well, which I think is a really great thing because then you’re not trying to make up a session.” [Faculty 3]

With respect to challenges, participants experienced difficulties with learner engagement and reported having little control over factors influencing decreased engagement in virtual settings. Distractions, the absence of computer cameras, and (un)muted microphones were mentioned most frequently. Nonetheless, participants emphasized that although virtual engagement was a challenge, it seldom compromised the objective and quality of learning. Participants attempted to overcome the challenges by using a variety of strategies to engage medical students and other residents throughout the virtual experience, including the use of visual media or props and asking questions.

“Some people… turn their cameras off… and then you probably know that they’re multitasking or something else in the background and not showing up in person and paying attention.” [Resident 4]

“My perception of it is that I think it is a little bit more challenging over Zoom to engage learners because it’s easy, I mean when you’re in a room and you know, everybody’s face to face… versus when everybody’s, you know, sitting in their own office or in their own room and they’re listening to somebody on a computer and there’s the option to have your camera on or off, or muted or not muted for your microphone.” [Resident 8]

“With the Zoom, there’s definitely times where there’s kind of distractions of course… a pet running up or kids running through, or you know, somebody’s not on mute and their partner walks by because they’re also working from home or whatnot.” [Resident 9]

### Impact on competence

Participants observed that teaching in virtual settings helped residents to overcome stage fright and shyness, with the added benefit of increased self-confidence with respect to teaching. Faculty also noted that they were better able to discreetly observe the residents teaching when faculty were joining individual breakout rooms. This resulted in fewer interruptions and residents felt less anxious while observed when teaching.

**Table 2:** Benefits and challenges of teaching in a virtual environment mapped to the SDT framework

SDT Framework – Basic psychological needs	Benefits (Facilitators)	Challenges (Barriers)
Autonomy	• Choice of virtual technology	• Lack of control over teaching environment
	• Increased accessibility	
	• Safety during pandemic	• Lack of control over factors affecting learner engagement
	• Save time and money	
Competence	• Increased self-confidence	• Technological limitations hindering teaching and skill development
	• Discreet observation of teaching	
Relatedness	• Timely exchange of information	• Limited personal connections and opportunities to socialize
	• Ease of sharing resources	• Difficulty with professional identify formation
	• Giving back to the profession	

“I get less stage fright. I think it disconnects you a little bit more from the audience members and you only worry about how you look from, you know, the waist up so … it’s reassuring, and you can do it in the comfort of your own home and sip on some coffee. I like that.” [Resident 1]

“When you’re kinda physically going from room to room watching…it is a bit disruptive for the residents and they feel maybe a little nervous when you pop in, but less so when you’re a virtual presence and just, a black screen pops in with your name as Observer. I think that the residents were much less flustered that way and less likely to stop their session to acknowledge that you’re there.” [Faculty 3]

With respect to challenges, insufficient technology and limited access to resources were repeatedly noted as a challenge to teaching and skill development in virtual settings. Participants reported that technology and access to resources often varied based on the geographical locations of residents and faculty. Participants mentioned non-functioning microphones, limited internet access, and lack of private teaching spaces as the most common technical limitations, which inadvertently hindered teaching and skill development.

“Definitely internet connection I thought was a barrier. So some people had like varying levels of internet connectivity and it would sometimes crash and make us spend extra time in the conference.” [Resident 2]

 “Residents who couldn’t leave their rotation sessions quickly enough… were forced to pull out their computer or tablet at the hospital in a relatively private section of a resident lounge or on one of the units in a room somewhere, but it was still quite noisy.” [Resident 7]

### Impact on relatedness

Residents and faculty indicated that one of the greatest benefits of the virtual platform was the timely exchange of helpful information to stay connected with the virtual community of learners and teachers. Participants often posted helpful tips and resources in the chat feature of virtual platforms during sessions. Many noted the ease of sharing teaching resources and a willingness to continue to utilize virtual resources post pandemic. Residents expressed enthusiasm to teach upon graduation and give back to the profession.

“I think maybe one of the positive things with Zoom is if someone says oh, I’ve got a great resource that I use for this and they can just send it in the chat, like send a PDF of the resource.” [Resident 9]

“One of the things I was really looking forward to as a resident is giving back the same way residents were really kind to me when I was a student.” [Resident 1]

Not surprisingly, residents described a lack of personal connections as opportunities to socialize with one another and foster feelings of comradery were limited in virtual settings. Throughout the COVID-19 pandemic, residents reported frequently seeking out other opportunities to stay connected and support one another. Faculty also noted difficulties with formation of professional identity for residents in virtual settings.

 “If we had to choose what our preference was I think most of us would’ve wanted to be in person, just because it’s a different social dynamic being there in person.” [Resident 7]

“…one of the huge benefits of the residents is yes, they do provide teaching, but they provide role modelling that then leads to at least an influence on professional identity formation, and virtually that’s really hard, or it’s harder to do, than it is when you compare it to doing it in-person.” [Faculty 9]

## Discussion

This study is novel in mapping the benefits and challenges of teaching in a virtual environment onto the SDT framework, and thereby identifying factors which have a potential to promote or undermine medical teachers’ needs for autonomy, competence, and relatedness. These innate psychological needs are critical for optimal functioning and wellbeing.^9^^-^^11^^,^^13^^,^^14^

With respect to autonomy, participants acknowledged that they enjoyed and benefited from increased accessibility, were motivated to think outside the box, and felt free to adapt their teaching strategies in the absence of a physical environment. The skills developed in a virtual environment were largely self-directed, and the payoff resulted in a greater understanding and expanded use of new technology, as well as a greater comfort with virtual technology in general. Overall, these findings are in line with SDT in that having autonomy to organize and carry out one’s work promotes and sustains intrinsic motivation, a beneficial form of motivation.^9^^-^^11^^,^^14^^-^^16^

In terms of competence, participants noted that having a mastery mindset was key to overcoming the challenges of virtual teaching such as technological issues and engaging learners effectively. Previous studies have demonstrated that having a mastery or growth mindset (vs. a fixed mindset) is associated with many desirable outcomes such as persistence, deep learning, self-efficacy (confidence) feelings, and having a healthy response to mistakes and setbacks, among others.^14^^,^^17^^-^^20^ In our study, this compelled study participants to try new resources and strategies for learner engagement, and to learn to be flexible while remaining focused despite distractions in the virtual environment.

As to the need for relatedness, participants acknowledged a variety of virtual platforms to choose from. They also noted that virtual platforms allow for a timely and frequent exchange of resources, thereby helping to stay connected and feel supported by the community of peers. Residents supported one another through self-organized virtual study sessions to overcome limited opportunities to socialize and to fulfill the need for relatedness, a key element in physician wellbeing.^21^^,^^22^ Despite limited personal connections in virtual settings, residents expressed enthusiasm to teach upon graduation and motivation to “pay it forward” to the professional community, which is in line with published research.^23^^-^^25^

### Limitations, future research, and implications

A limitation of our study is that it examined perspectives of residents and faculty in a single residency program. Participants in this study were primarily females in the urban stream, as such, the findings may not be representative of males and of residents and faculty in the rural stream or those in other disciplines. Hence, a larger study examining facilitators and barriers to virtual teaching in a wider range of specialties is warranted.

The SDT-informed teaching strategies shown to be effective in traditional in-person teaching settings^16^ need to be examined for their applicability in virtual settings. Some of the recommendations for the future delivery of virtual teaching include: giving high priority to engagement and active participation; nurturing autonomy and greater individual responsibility for learning; and creating an environment of emotional support. Empirical research in this area is timely and highly needed.

What may be perceived to be a challenge to some, may be a benefit/facilitator to others (e.g., technology, home vs. work environment). As such, suggestions for future development for novice teachers should include best practices to optimize the online experience and support future physicians in their teaching roles. Our findings demonstrate the importance of considering both the resident and faculty perspectives. Novice and experienced teachers can enrich the virtual experience by helping one another overcome constraints, share resources, and optimize practice scenarios, especially for those working in rural and remote regions, and thereby, creating a virtual community of practice.^23^

Our findings indicate that virtual settings appear to afford a conducive environment for residents to develop self-confidence as a first step to teaching and for faculty to support residents by directly observing them teach, with minimal interruptions to the teaching process. Additionally, faculty noted the potential impediment of virtual settings on residents’ professional identify formation. As such, quantitative (e.g., longitudinal) and qualitative studies investigating the use of virtual technology to help novice teachers develop self-confidence and internalize professional attitudes, values, beliefs, and skills are warranted.

## Conclusions

Residents are future faculty who require effective teaching skills in the digital era. Employing the SDT framework, we identified how residents and faculty used technology not only to deliver education but also to help support their psychological needs for autonomy, competence, and relatedness in virtual settings. The key challenges appear to be technology problems and difficulties with participant engagement in virtual teaching environments. Some technological challenges may be easier to address, while others are external and beyond the scope of residency programs. Virtual platforms are one venue in which physicians will likely continue to teach in the future, and residency programs should address the challenges of virtual teaching in order to fulfill the psychological needs and professional identity of medical educators.

### Conflict of Interest

The authors declare that they have no conflict of interest.

## Supplementary material

Supplementary file 1 Appendix. Interview question
