# Supplementary material for: Psychological need fulfillment in virtual teaching: insights of residents and faculty
Source: Int J Med Educ. 2023 Jun 22;14:77–83. doi: 10.5116/ijme.6488.2625 (PMC10693391; doi:10.5116/ijme.6488.2625)
Supplement: Supplementary file 1 — Appendix. Interview question [file ijme-14-77-S1.pdf]

Appendix

Interview question

Specifically, describe the VIRTUAL teaching opportunities (formal/informal) in greater detail.

Prompts:

- What virtual platform was used? (e.g., Zoom, Google Meets, Podcasts, YouTube, TikTok)
- What preparation did you need to do? (e.g., technology, camera set-up, visuals, PowerPoint)
- What communication styles did you use? (e.g., models, videos, drawing, documents)
- Was it easier or more challenging to communicate, and why?
- Was it different than in-person teaching?
- Was it easier to prepare or more challenging, and why?
- What did to you do to engage the learners?
- Was it easier or more challenging to engage, and why?
- Did you encounter anything you would not normally in person (e.g., kids, pets, environment)? Were these encounters helpful or distracting?
- What differences did you note between virtual and in person teaching?
- What were some barriers you encountered and how did you overcome them?
- What benefits have you seen from virtual teaching?

Note: While various prompts were prepared for this question in advance, most responses pertained to the barriers and challenges of virtual teaching and are the focus of this analysis.
